# The development and validation of a questionnaire to assess female athletes’ and coaches’ knowledge about hormonal contraception

**DOI:** 10.3389/fspor.2026.1921305

**Published:** 2026-10-23

**Authors:** Katharina Fischer, Elisabeth Maria Kirschbaum, Lucie Nikoleizig, Anne-Marie Elbe, Kerry McGawley

**Affiliations:** 1 Department of Sport Psychology, Sport Science Faculty, Leipzig University, Leipzig, Germany; 2 Research Group Elite Female Athletes, Institute for Applied Training Science, Leipzig, Germany; 3 Movement Biomechanics, Institute of Sport Science, Humboldt-Universität zu Berlin, Berlin, Germany; 4 Faculty of Culture, Media & Psychology, Macromedia University of Applied Science, Leipzig, Germany; 5 Swedish Winter Sports Research Centre, Department of Health Sciences, Mid Sweden University, Östersund, Sweden

**Keywords:** competitive sport, instrument development, knowledge test, menstrual health, oral contraceptives, questionnaire construction

## Abstract

**Introduction:**

In this study, a hormonal contraception knowledge questionnaire (HCKQ) for female athletes and coaches was developed and validated.

**Methods:**

An initial 17-item questionnaire (Pre-HCKQ), including items adapted from existing tools and newly generated items, was piloted with 325 recreational female athletes and 46 coaches. The analysis of item difficulty index and item-total correlation resulted in a 10-item HCKQ, which included questions about the safety of hormonal contraceptive (HC) use, function and effects of different HCs, risk factors and side effects of HC use, and the fertile window in the menstrual cycle. Eighteen experts evaluated the HCKQ for relevance (*M* = 5.5, *SD* = 0.8) and clarity (*M* = 5.5, *SD* = 0.9) using a 6-point Likert scale.

**Results:**

The HCKQ was subsequently administered to 562 elite female athletes and 170 coaches. The mean difficulty index was 46%, and the mean item-total correlation was *r* = .29. A significant difference in knowledge scores was found between female athletes, female coaches, and male coaches, as well as between HC users and non-users. Reliability was assessed through internal consistency (Cronbach's *α* = .61, McDonald's *ω* = .62, Guttman's *λ*₂ = .63) and test-retest in a separate, mixed-sex cohort of 64 sport science students (Spearman's *ρ* = .75; ICC(2,1) = .74).

**Discussion:**

The HCKQ provides a brief tool for assessing HC-related knowledge in sports contexts, with initial evidence supporting its content validity, known-groups validity, and reliability. Future research may use the HCKQ to evaluate the effectiveness of female health education programs in sport.

## 1 Introduction

Female athletes train and compete under the influence of fluctuating reproductive or synthetic hormones, whether due to their menstrual cycles or the use of hormonal contraceptives (HCs). Despite widespread use of HCs among female athletes (e.g., 1, 2), knowledge within athlete and coach populations appears limited in relation to the different types of HCs available, their mechanisms, and implications for health and performance (e.g., 3, 4). Such foundational knowledge of HCs may support athletes and coaches in asking informed questions, recognizing relevant knowledge gaps, and engaging in shared decision-making with qualified healthcare professionals. Elite sport in Germany is characterized by a federal and federation-based support system, whereby support for athletes and coaches is provided through various stakeholders and locations, including Olympic training centers. Although Olympic training centers play a central role in medical and interdisciplinary support, current analyses indicate heterogeneous quality and implementation of existing support structures (5, 6). This may influence the extent to which athletes and coaches have access to specific information on female health in sport, including HCs.

HCs primarily contain synthetic progestins and/or estrogens, which modify the body's natural hormonal milieu by inhibiting endogenous hormone production. This ultimately prevents pregnancy through a variety of mechanisms. Combined progestin-estrogen methods primarily suppress the secretion of luteinizing hormone (LH) and follicle-stimulating hormone (FSH), thereby inhibiting ovulation. Progestin-only methods vary in their effects on ovulation; some formulations may not consistently inhibit ovulation but instead act mainly by thickening the cervical mucus to block sperm penetration. In addition, many HCs reduce fallopian tube motility and limit endometrial development to make implantation less likely (7). The specific effects of HCs can further vary depending on the formulation (i.e., combined or progestin-only), route of application (oral, intrauterine, or implant), and product brand (8, 9).

The prevalence of HC use among female athletes varies widely, ranging from approximately 30% to 68%, depending on individual characteristics, sport type, and personal motives for use (1, 2, 10–13). HC use appears to be more common among female athletes who are older and are in a relationship (12). Recent public health data suggest a potential generational shift in contraceptive behavior, reflected in declining use of oral contraceptive pills (OCPs) among young people aged 16–25 years in Germany (14). This trend may be related to increasingly critical attitudes toward HCs, including concerns about side effects and a desire to avoid or discontinue exogenous hormone use (15, 16). Reported concerns relating to HC use commonly include headaches, weight gain, mood changes, and reduced libido (16). Although evidence specific to athletes remains limited, these broader generational shifts may also have implications for HC use patterns within athletic populations.

Besides contraceptive purposes, female athletes use HCs for additional benefits, such as managing menstrual symptoms and cycle timing, regulating menstrual bleeding to better align with training and competition schedules, reducing menstrual cramps and heavy menstrual bleeding, and improving skin quality (2, 10–13). However, HCs are also associated with negative side effects, including mood swings (10, 17, 18), weight gain (13), tiredness, bloating, and abdominal pain (2, 11). Regarding the perceived effects of HC use on performance, Baumgartner et al. (1) reported that 31.5% of 181 Swiss female elite athletes perceived no noticeable impact, 43.6% were unsure, and a minority reported benefits such as more consistent performance (14.4%) or feeling more powerful (7.7%).

Foundational knowledge of HC types, composition, and effects may help female athletes to ask informed questions and engage in shared decision-making with qualified healthcare professionals, particularly in relation to individual health, well-being, and performance-related goals (4). Consulting a gynecologist is important when making these decisions, to account for factors such as genetic risk (e.g., thrombosis; 19). Ideally, the contraception method should be simple to use, pose minimal risks and negative side effects, and offer the potential for additional benefits, such as reducing menstrual discomfort and/or heavy menstrual bleeding (17). Given their specific physical performance and lifestyle needs, elite athletes require sport-specific HC advice, which may exceed the expertise of most gynecologists. To address this issue, several German-speaking countries have established contact lists to facilitate athletes’ access to gynecologists with sport-specific expertise (for example: 20–22). Equally importantly, female athletes and their coaches need foundational knowledge of the available types, mechanisms, and effects of HCs to inform decision making, especially in this often young (athlete) population (23).

Two studies to date have examined OCP knowledge among female athletes (4) and coaches (3), each using their own developed questionnaires. These studies made important contributions to the scientific literature by providing initial insights into the degree to which female athletes and coaches understand OCPs. Mean knowledge scores were 36% for female athletes, 40% for female coaches, and 28% for male coaches, with a significant difference observed between female and male coaches. Furthermore, female athlete HC users demonstrated a significantly better understanding of OCPs compared to non-users (4). Both studies focused only on OCPs, while other HC types and underlying mechanisms were not examined. The questionnaires used in these studies also had some methodological limitations. For example, Larsen et al. (4) did not report item-level psychometric indicators, such as item difficulty or item discrimination, which limits conclusions about the psychometric performance of individual items. Johnson's (3) 12-item tool, in contrast, used mixed-answer formats, including open-ended items, which may complicate scoring and reduce comparability across respondents (24). In addition, previous tools have typically focused on either athletes or coaches, rather than including both populations.

More recently, Larsen et al. (25) developed an updated questionnaire assessing both menstrual cycle and HC knowledge among athletes and sports performance support staff. While this updated instrument provides a broader assessment of menstrual cycle and HC knowledge, the HC knowledge questionnaire (HCKQ) proposed in the present study offers a targeted tool for assessing foundational HC knowledge in sport populations, including female athletes and coaches, and includes item-level psychometric properties. Unlike previous tools, the HCKQ will incorporate items on intrauterine systems (IUSs), a method used as frequently as OCPs among elite female athletes in Germany (12). Furthermore, the HCKQ was designed with input from gynecological experts to ensure both scientific and practical relevance. Based on previous evidence showing group differences in mean knowledge scores, defined as the number or percentage of correctly answered knowledge items (3, 4), we hypothesized that female coaches would score higher on the HCKQ than male coaches, and that HC users would outperform non-HC users.

## 2 Methods

### 2.1 Ethics approval and consent to participate

This study was approved by the Ethics Committee of the Institute for Applied Training Science, Leipzig, Germany (ER_2021.22.06_7, ER_2022.01.17_12). Participants provided informed consent electronically after receiving information about the questionnaire topics, procedures, data protection, and confidentiality. Written parental consent was obtained for female athletes under the age of 16 years.

### 2.2 Study overview

The development and validation of the HCKQ was guided by the studies of Boateng et al. (26) and Parmenter and Wardle (27) and involved a nine-step process (see the sub-section below, “*Nine-step development and validation process*”, for more details), which was organized into three phases (Figure 1), as per Fischer et al. (28): item generation (steps 1–3), questionnaire development (steps 4–5), and questionnaire evaluation (steps 6–9). All online questionnaires in steps 4–9 were administered using SoSci Survey (29). The HCKQ was developed and validated in German and an English translation was prepared by bilingual and native German and English co-authors for international publication purposes only (see Table 4). This translation is not a validated English-language version of the HCKQ.

**Figure 1:**
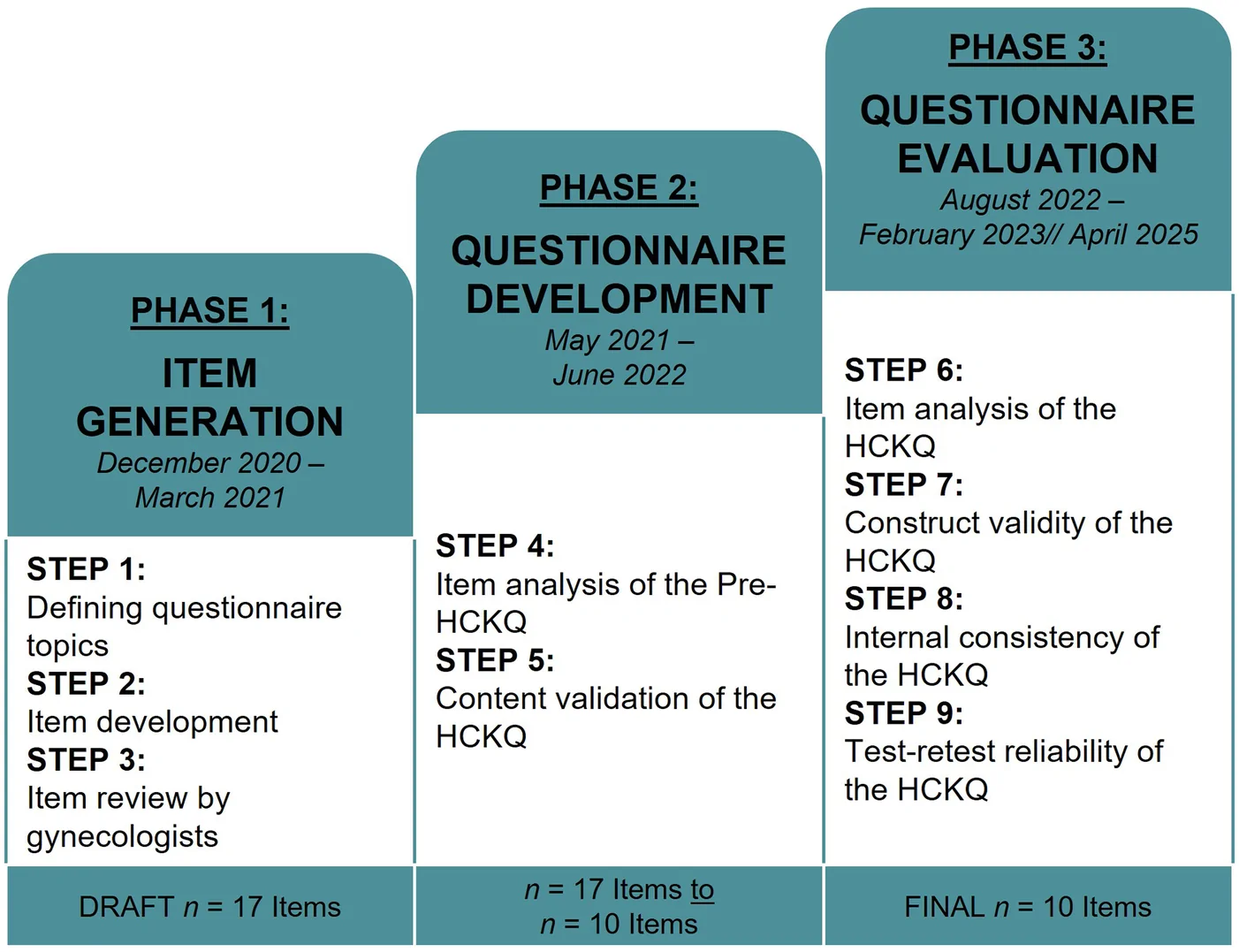
An overview of the three phases (9 steps) of developing and validating the 10-item hormonal contraception knowledge questionnaire (HCKQ).

### 2.3 Participants and recruitment

Different participant groups contributed to different stages of the nine-step development and validation process and are therefore described below according to the step(s) in which they were involved.

#### 2.3.1 Steps 2–3: Gynecological experts for item development and item review

In steps 2–3, three gynecologists (2 females, 1 male; professional experience 3–17 years) were recruited from the authors’ networks between December 2020 and March 2021.

#### 2.3.2 Step 4: Female recreational athletes and coaches for pre-HCKQ item analysis

In step 4, female recreational athletes (*n* = 325; age: *M* = 25.5, *SD* = 7.1 years) and coaches (*n* = 46; 27 females [59%], 19 males [41%]; age: *M* = 28.2, *SD* = 6.9 years) were recruited between May and July 2021 via various channels, including WhatsApp, sports clubs, e-mails, social media platforms (particularly sports groups on Facebook), and posters at Leipzig University's sports science campus. Participants were ≥ 16 years, and elite athletes were excluded from this step.

#### 2.3.3 Step 5: Expert panel for content validation

In step 5, 18 experts (17 female, 1 male; age: *M* = 38.8, *SD* = 11.4 years) were invited through the authors’ networks to form an expert panel to review the HCKQ. The panel included seven gynecologists with applied expertise in gynecological health, five biologists with relevant academic expertise, four sports physicians with applied expertise in athlete health and medical care, and two sports scientists with applied experience and a particular interest in gynecological health in sport.

#### 2.3.4 Steps 6–8: Elite female athletes and coaches for questionnaire evaluation

In steps 6–8, 562 German elite female athletes (age: *M* = 21.0, *SD* = 5.1 years) and 170 coaches of elite female athletes (55 female [32%], 114 male [67%], 1 unspecified; age: *M* = 37.5, *SD* = 10.9 years) were recruited through national sports federations, Germany's independent athletes’ association (Athleten Deutschland e.V.), the Cologne coach academy of the German Olympic sports federation (Trainerakademie Köln des Deutschen Olympischen Sportbundes e.V.), the professional association of coaches in Germany (Berufsverband für Trainer/innen im deutschen Sport), and via social media (e.g., Facebook and Instagram channels of the Institute for Applied Training Science, Leipzig, Germany [Institut für Angewandte Trainingswissenschaft]). Data collection occurred between August 2022 and February 2023.

#### 2.3.5 Step 9: Sport science students for test-retest reliability

In step 9, 64 sport science students at Leipzig University (34 female [53%], 29 male [45%], 1 unspecified; age: *M* = 21.3, *SD* = 1.7 years) were invited to complete the questionnaire twice in April 2025.

### 2.4 Nine-step development and validation process

#### 2.4.1 Phase 1: Item generation (steps 1–3)

Step 1—Defining questionnaire topics: A literature review was conducted by the first three authors to define the construct of HC knowledge and to identify relevant content domains for the questionnaire. Scientific literature and non-scientific educational materials on HC were reviewed to identify recurring topics related to the use, effects, benefits, risks, and reproductive implications of HCs. Although the review was not conducted as a systematic review, it followed a structured process whereby relevant information was extracted from the selected sources, discussed among the authors, and synthesized into preliminary content domains.

Step 2—Item development: The first and second authors reviewed existing questionnaires and relevant publications in English and German to identify items suitable for inclusion in the initial version of the questionnaire (Pre-HCKQ). In addition, the gynecologists suggested further items based on their clinical expertise. Items were evaluated for content relevance, and overlapping items were combined to form coherent item groups, resulting in a preliminary structure for the questionnaire. Items were formulated to assess one knowledge aspect at a time, to avoid double-barreled wording, and using clear and concise language. Response options were designed to include one clearly correct answer and a “do not know” option to reduce guessing.

Step 3—Item review by gynecologists: The Pre-HCKQ underwent a detailed review by the gynecologists using an online discussion format. The review followed a structured item-by-item procedure, in which each item was assessed for content accuracy, clinical relevance, and language clarity. Items were revised where necessary, and the agreed revisions were incorporated into the subsequent version of the questionnaire.

#### 2.4.2 Phase 2: Questionnaire development (steps 4–5)

Step 4—Item analysis of the Pre-HCKQ: All preliminary items were evaluated to determine which should be retained in the final questionnaire (HCKQ). Item difficulty (i.e., percentages of correct answers for each question) and item-total correlation (i.e., point-biserial correlations between the score for each question and the total score) were calculated. Items meeting at least one of the predefined criteria (see the “*Statistical analys*e*s”* sub-section below for more details) were retained, resulting in a pool of 10 items.

Step 5—Content validation of the HCKQ: Eighteen experts rated each of the 10 items for clarity and relevance. These ratings were used to calculate the item-level and scale-level content validity index (CVI) to quantify agreement (see the sub-section below, “*Statistical analyses”*, for more details). In addition, a video conference with five of the experts was held to refine the wording of the items and to reach consensus on any necessary revisions.

#### 2.4.3 Phase 3: Questionnaire evaluation (steps 6–9)

Step 6—Item analysis of the HCKQ: The HCKQ was distributed to the 562 elite female athletes and 170 coaches. Item difficulty and item-total correlations were recalculated to confirm the performance of each item.

Step 7—Construct validity of the HCKQ: Construct validity was assessed by testing whether HCKQ scores could distinguish between the groups, which were hypothesized to differ in HC knowledge.

Step 8—Internal consistency of the HCKQ: Cronbach's *α* was calculated to assess internal consistency. McDonald's *ω* and Guttman's *λ*₂ were also computed, to provide more suitable reliability estimates when the assumptions of tau-equivalence and normality are violated.

Step 9—Test-retest reliability of the HCKQ: To assess stability over time, 64 sport science students completed the HCKQ twice, with a one-week interval between administrations (30). Sport science students were recruited because they represented an accessible population with sport-related educational backgrounds and familiarity with sport contexts. Therefore, this group was considered appropriate for estimating the questionnaire's temporal stability. However, specific data on this group's athletic or coaching backgrounds were not collected.

### 2.5 Sample size calculations

The sample size calculation for step 4 (item analysis of the Pre-HCKQ) followed the “rule of thumb” proposed by Rosi et al. (31), which recommends a minimum ratio of 10 participants per questionnaire item to ensure representation of the intended population. With 17 items in the questionnaire, the minimum target sample size was 170 participants. Efforts were made to recruit a diverse group of recreational female athletes and coaches. Recreational athletes were defined as those who regularly participated in sports, and coaches were individuals working voluntarily for a sports club. Our sample sizes of 325 athletes and 46 coaches exceeded the minimum limit more than twofold. For step 5 (content validation of the HCKQ), as recommended by Haynes et al. (32), we included at least five experts in women's sports and gynecological health. Including a larger number of experts further enhances the precision and reliability of the ratings, reduces the standard error of measurement, and strengthens confidence in the content validation process. Our sample of 18 experts fulfilled these requirements. For steps 6–8 (item analysis, internal consistency and construct validity of the HCKQ), sample size calculations were performed using G*Power (Version 3.1.9.7, Düsseldorf, Germany; 33). An *a priori t*-test for the difference of means between two groups (i.e., HC users vs. non-HC users) was used to determine the required sample size for the elite female athlete group. The power analysis was based on Larsen et al. (4), who reported an average HC knowledge score of *M* = 5.03 (*SD* = 3.06; maximum score = 14), with HC users scoring on average 1.79 points higher than non-HC users. This corresponded to an effect size of *d* = .33, which was considered meaningful for distinguishing between groups with differing HC knowledge. Assuming an alpha level of .05 and a power of 80%, the required sample size for this study was 196 participants per group (HC users vs. non-HC users). For the coach sample, the power analysis was based on Johnson (3), who reported average HC knowledge scores of *M* = 40.1% (*SD* = 14.7%) and *M* = 27.6% (*SD* = 13.7%) for female and male coaches, respectively (maximum score = 38). This difference corresponded to an effect size of *d* = .96. Using the same alpha level and power, the required sample size for this study was 59 participants per group.

### 2.6 Statistical analyses

Item difficulty was calculated as the percentage of participants answering each item correctly. According to Bauhaus et al. (34), items with difficulty indices between 10% and 90% should generally be retained. Items outside of this range, as well as items close to the upper and lower boundaries, were reviewed for potential rephrasing or elimination to ensure an appropriate level of difficulty for the target population.

Item-total correlations were computed to evaluate each item's contribution to overall scale consistency. The point-biserial correlation coefficient (mathematically equivalent to the Pearson product-moment correlation when one variable is dichotomous) was used. A correlation of *r* ≥ .20 was considered the minimum acceptable criterion, whereas *r* ≥ .30 was used as the preferred criterion for good item discrimination. Item-total correlations were categorized as *r* < .20 = poor, *r* = .20–.29 = acceptable, *r* = .30–.39 = good, and *r* ≥ .40 = excellent (35). Items essential for content validity were allowed to meet a lower minimum threshold of *r* ≥ .10, as described by Garrett and Woodworth (36). As a first step, items with *r* values < .30 were reviewed by an expert panel (i.e., the three gynecologists involved in steps 2–3 and the first two authors) and items judged to be important for content validity were retained, whilst others were removed.

Content validity was assessed using the CVI, following established guidelines (37, 38). Each item was rated for relevance (“not at all relevant” = 1 to “very relevant” = 6) and clarity (“not at all clear” = 1 to “very clear” = 6) on a 6-point Likert scale. The item-level CVI (I-CVI) was calculated as the proportion of experts rating an item 4–6. I-CVI values ≥ .79 were considered acceptable, items scoring .70–.79 were modified, and items < .70 were eliminated (39). The scale-level CVI (S-CVI) was computed as the mean of all I-CVI values.

Differences in knowledge scores between the female athletes, female coaches, and male coaches were examined using a one-way ANOVA with Bonferroni *post-hoc* tests. Effect sizes were interpreted as: *f* ≥ .10 = small, *f* ≥ .25 = medium, or *f* ≥ .40 = large (40). Differences between HC users and non-HC users were assessed using an independent-samples Welch's *t*-test, with effect sizes interpreted as: *r* ≥ .10 = small, *r* ≥ .30 = medium, or *r* ≥ .50 = large (41).

The internal consistency reliability of the scale was assessed using Cronbach's *α*, complemented by McDonald's *ω* and Guttman's *λ*₂. Although a Cronbach's *α* of ≥ .70 is generally considered the lower limit for acceptable internal consistency, a threshold of .60 may be considered appropriate in exploratory research (42). As the HCKQ is a newly developed instrument undergoing initial validation, reliability coefficients between .60 and .70 were interpreted as indicating modest but acceptable internal consistency for this stage of scale development.

For the test-retest analysis, Bland-Altman plots and paired-samples *t*-tests were first used to examine measurement agreement and detect systematic bias in the knowledge score (43). Test-retest reliability was then evaluated using Spearman's correlation coefficients and intraclass correlation coefficients (ICCs) from a two-way mixed-effects model with absolute agreement (43). Spearman's *ρ* was interpreted as < .20 = very weak, < .40 = weak, < .70 = moderate, < .90 = strong, and ≥ .90 = very strong (44). ICC values were interpreted as < .50 = poor, < .75 = moderate, < .90 = good, and > .90 = excellent reliability (45).

All data analyzes were conducted using IBM SPSS Statistics for Windows (Versions 23 and 29 [with internal consistency reliability analyses conducted in Version 29]; IBM Corp., Armonk, NY, USA) and Excel (version 2016, Microsoft Corp., Redmond, WA, USA). Results are presented as means (*M*) with standard deviations (*SD*), percentages (e.g., item difficulty), or frequencies. Statistical significance was set at *p* < .05 for all analyses.

## 3 Results

### 3.1 Phase 1: Item generation

In step 1 (defining questionnaire topics), HC knowledge was defined as knowledge about hormonal methods available for preventing unintended pregnancy, including their mechanisms of action, safety, potential benefits and risks, side effects, and implications for the menstrual cycle and fertility. Based on this construct definition and the reviewed materials, four content domains were identified for the questionnaire: safety of HC use, functions and effects of different HC methods, risk factors and side effects, and the fertile window within the menstrual cycle.

In step 2 (item development), questionnaire items were developed using two complementary approaches. In the first approach, the three gynecologists suggested 39 potential items. Of these, 11 items were excluded because they related to intake protocols and use of OCPs (*n* = 6), or comparisons with other contraceptive methods (*n* = 5). The remaining 28 items were consolidated by merging 13 overlapping items, resulting in 15 item groups. In the second approach, 32 items were identified from existing questionnaires; 14 were excluded (12 on intake protocols and OCP use, 2 on knowledge and attitudes toward HCs), and the remaining 18 items were integrated into the 15-item groups proposed by the gynecologists. Four of these groups included multiple items due to thematic overlap, and one additional item was introduced as a distinct new group (see Supplementary Table S1).

In step 3 (item review by gynecologists), each item was refined to include four single-choice response options, either formulated by the gynecologists or adapted from existing literature, plus a “do not know” option. For two items, three correct response options were possible, and participants could select “all of the above” as the correct answer. The final process in this step involved a discussion among the gynecologists and the first three authors, which led to the addition of one further item group. The final structure of the questionnaire included 17 Pre-HCKQ items covering safety of HC use (*n* = 3), mechanisms of HC actions and effects (*n* = 8), risk factors and side effects of HC use (*n* = 3), and the fertile window in the menstrual cycle (*n* = 3).

### 3.2 Phase 2: Questionnaire development

The step 4 (item analysis Pre-HCKQ) item difficulty and item-total correlation results are presented in Table 1. One item (HC_01), correctly answered by 90% of respondents, was removed because it was at the upper boundary of the predefined difficulty range and provided limited discriminatory value. Four additional items were also excluded because their item-total correlations were below the preferred criterion of *r* ≥ .30 (HC_02, HC_12, HC_15, HC_17). Several other items with similarly low item-total correlation (i.e., *r* < .30) were retained based on expert judgment. These items were considered essential for content validity as they addressed safety of HC use (HC_03), fertility within the menstrual cycle (HC_04), a simple introductory “icebreaker” item (HC_06), use of OCPs to prevent withdrawal bleeding (HC_10; (1)), and hormones contained in IUSs (HC_11). The two items with the response option “all of the above” were removed (HC_09, HC_16), as they were judged likely to promote guessing rather than accurately assessing knowledge. Excluding these seven items resulted in a 10-item HCKQ.

**Table 1:** Item descriptions, difficulty index, item-total correlation scores, and reasons for item exclusion for the initial 17-item hormonal contraception knowledge questionnaire (pre-HCKQ). Items were translated to English (from German), and data are based on responses from *n* = 371 female athletes and coaches.

Item no.	Item	Item difficulty index [%]	*SD* [%]	Item-total correlation	Exclusion
HC_01	When are the fertile days in a menstrual cycle?	0.90	0.31	0.22	Item difficulty
HC_02	How long are sperm viable?	0.50	0.50	0.24	Item-total correlation
HC_03*	**Which of the following contraceptive method is the safest?**	**0**.**65**	**0**.**48**	**0**.**14**	
HC_04*	**How long does an egg stay fertile?**	**0**.**28**	**0**.**45**	**0**.**20**	
HC_05*	**What does the PEARL index mean?**	**0**.**53**	**0**.**50**	**0**.**44**	
HC_06*	**What does not influence the effect of the pill?**	**0**.**81**	**0**.**39**	**0**.**21**	
HC_07*	**Which two hormones are in most birth control pills?**	**0**.**70**	**0**.**46**	**0**.**41**	
HC_08*	**What is not an advantage of hormonal contraceptive methods?**	**0**.**84**	**0**.**37**	**0**.**35**	
HC_09	What are the most common side effects of the pill?	0.77	0.42	0.33	Response format
HC_10*	**What can happen when using the pill as a hormonal contraceptive if pills without active ingredients are skipped and pills with active ingredients are taken continuously?**	**0**.**66**	**0**.**48**	**0**.**27**	
HC_11*	**Which hormones are in a hormonal IUS?**	**0**.**17**	**0**.**38**	**0**.**27**	
HC_12	How long does a hormonal IUS work on average?	0.71	0.45	0.24	Item-total correlation
HC_13*	**Which statement about the effect of the pill is false?**	**0**.**53**	**0**.**50**	**0**.**31**	
HC_14*	**How does a hormonal IUS work?**	**0**.**27**	**0**.**45**	**0**.**31**	
HC_15	What is a risk factor for taking the pill?	0.68	0.47	0.22	Item-total correlation
HC_16	What distinguishes the minipill from other monophasic and multiphasic birth control pills?	0.25	0.43	0.33	Response format
HC_17	Which classification of the effectiveness of different contraceptives (starting with the least safe) is correct?	0.34	0.48	0.11	Item-total correlation

*Items in bold were retained and included in the final 10-item HCKQ.

*SD*, standard deviation; IUS, Intrauterine System.

In step 5 (content validation of the HCKQ), the panel of 18 experts rated the 10-item HCKQ as relevant (*M* = 5.5, *SD* = 0.80) and clear (*M* = 5.5, *SD* = 0.90; see Supplementary Table S2 for detailed results). The I-CVI ranged from .89 to 1.00 and the S-CVI was 97% (Table 2). Linguistic refinements were made during a video conference with five members of the 18-person expert panel, who represented gynecological and sport-related expertise. Refinements included rephrasing items and response options to improve clarity and comprehensibility; specific changes are documented in the supporting information, Supplementary Table S3.

**Table 2:** Item content validity index (I-CVI) and scale content validity index (S-CVI) scores for the hormonal contraception knowledge questionnaire (HCKQ) items as rated by *n* = 18 experts on a scale of 1 (not relevant at all) to 6 (very relevant). .

Item no.	Ex-pert 1	Ex-pert 2	Ex-pert 3	Ex-pert 4	Ex-pert 5	Ex-pert 6	Ex-pert 7	Ex-pert 8	Ex-pert 9	Ex-pert 10	Ex-pert 11	Ex-pert 12	Ex-pert 13	Ex-pert 14	Ex-pert 15	Ex-pert 16	Ex-pert 17	Ex-pert 18	*Experts in agree-ment (n)*	I-CVI
HC_3	6	6	5	6	5	6	6	6	5	6	6	6	6	6	6	6	6	4	18	1.00
HC_4	6	6	5	5	5	6	5	6	4	4	6	6	6	6	6	6	6	3	17	0.94
HC_5	6	6	5	5	5	6	5	3	5	6	6	6	6	6	6	6	6	2	16	0.89
HC_6	4	6	5	6	6	6	6	6	5	3	6	6	6	6	6	6	6	3	16	0.89
HC_7	5	6	5	4	6	6	5	6	5	6	6	6	6	6	6	6	6	4	18	1.00
HC_8	4	6	5	5	5	6	6	6	5	4	6	6	6	6	6	6	5	4	18	1.00
HC_10	6	6	4	6	5	6	6	6	5	6	6	6	6	6	6	5	6	4	18	1.00
HC_11	6	6	5	4	5	6	5	4	5	3	6	6	6	6	6	6	5	4	17	0.94
HC_13	6	6	5	5	6	6	5	6	5	4	6	6	6	5	6	6	6	4	18	1.00
HC_14	6	6	5	5	5	6	4	6	5	6	6	6	6	6	6	6	6	4	18	1.00
S-CVI	1,00	1,00	1,00	1,00	1,00	1,00	1,00	0,90	1,00	0,80	1,00	1,00	1,00	1,00	1,00	1,00	1,00	0,70		0.97

The I-CVI represents the proportion of experts rating an item 4–6. The S-CVI is the mean of all I-CVI values.

### 3.3 Phase 3: Questionnaire evaluation

The step 6 (item analysis) participant information for the female athletes and coaches who completed the 10-item HCKQ is presented in Table 3. The HCKQ with response options (presented in English [translated] and German [original]), as well as item difficulty index and item-total correlation, are presented in Table 4. The mean difficulty index was *M* = 46% (*SD* = 26%). Item HC_06 was the easiest for the participants to answer (88%), and item HC_11 was the most difficult (8%). The mean item-total correlation was *r* = .29 (*SD* = .09; range = .14 –.46). The item-total correlation was poor for *n* = 1, acceptable for *n* = 6, good for *n* = 1, and excellent for *n* = 2.

**Table 3:** Descriptive (including demographic and educational) information for the elite female athletes, and the female and male coaches who completed the hormonal contraception knowledge questionnaire (HCKQ).

Characteristics	Female athletes (*n* = 562)	Female coaches (*n* = 55)	Male coaches (*n* = 114)
*M* ± *SD* (*min–max*)			
Age (years)	21.0 ± 5.1 (14–43)	34.0 ± 10.7 (18–65)	39.3 ± 10.6 (19–70)
Menarche age (years, *n* = 544)	13.4 ± 1.6 (9–19)		
Weekly training (hours)	17.6 ± 7.3 (5–45)		
Years in primary sport (athletes) or coaching experience (coaches)	11.1 ± 5.1 (0–35)	10.7 ± 9.6 (1–40)	13.8 ± 9.0 (1–38)
Average age of female athlete training group (years)		15.7 ± 4.0 (7–25)	19.9 ± 4.7 (12–35)
*n* (%)			
HC user	162 (28.8)		
Highest educational level			
No school-leaving qualification	74 (13.2)		
9th grade	13 (2.3)		2 (1.7)
10th grade	101 (18.0)	3 (5.5)	6 (5.3)
12th/13th grade	259 (46.1)	10 (18.2)	19 (16.7)
Apprenticeship	16 (2.8)	2 (3.6)	5 (4.4)
Polytechnic degree	12 (2.1)	2 (3.6)	4 (3.5)
Bachelor, Master	85 (15.1)	37 (67.3)	75 (65.8)
Doctorate (PhD)	2 (0.4)	1 (1.8)	3 (2.6)
Competition level			
International	408 (72.6)		
National	130 (23.1)		
Regional	7 (1.3)		
Other	17 (3.0)		
Sport discipline			
Endurance	180 (32.0)	30 (54.6)	51 (44.7)
Power	78 (13.9)	8 (14.5)	29 (25.4)
Aesthetic	45 (8.0)	4 (7.3)	
Combat	85 (15.1)	4 (7.3)	10 (8.8)
Ball Games	123 (21.9)	8 (14.5)	22 (19.3)
Other	51 (9.1)	1 (1.8)	2 (1.8)

The coach with sex unspecified (*n* = 1) is not listed in the table to protect anonymity.

*M*, mean; *SD*, standard deviation; *min*, minimum; *max*, maximum; HC, hormonal contraceptive.

**Table 4:** Item descriptions (with English translations and the original German version), difficulty index and item-total correlation scores for the final 10-item hormonal contraception knowledge questionnaire (HCKQ).

Item no.	Item (English)	Item (German)	Item difficulty index [%]	*SD* [%]	Item-total correlation
HC_3	Which of the following contraceptive method is the most effective according to the PEARL index? − Oral contraceptive pill − Condom − **Hormonal IUS** − Calendar method	Welche der nachfolgenden Verhütungsmethoden ist die sicherste gemäß dem PEARL-Index? − Verhütungspille − Kondom − **Hormonspirale** − Kalendermethode	0.26	0.44	0.14
HC_4	Sperm can survive for up to 5 days in the female genital tract. How long is an egg fertile? − approx. 8 h − **approx. 1 day** − approx. 3–4 days − approx. 5–8 days	Spermien können bis zu 5 Tage im weiblichen Genitaltrakt überleben. Wie lange ist eine Eizelle befruchtungsfähig? − ca. 8 Stunden − **ca. 1 Tag** − ca. 3–4 Tage − ca. 5–8 Tage	0.26	0.44	0.22
HC_5	What is the PEARL index? − Measurement of a woman's fertility − Measurement of how many menstrual cycles a woman will have in her lifetime − **Measurement of the reliability of a contraceptive method** − Measurement of the composition of hormones in the contraceptive pill	Was gibt der PEARL-Index an? − Maß für die Fruchtbarkeit der Frau − Maß dafür. wie viele Menstruationszyklen eine Frau in ihrem Leben haben wird − **Maß für die Zuverlässigkeit einer Verhütungsmethode** − Maß für die Zusammensetzung der Hormone in der Verhütungspille	0.43	0.50	0.46
HC_6	Which factor does not generally influence the effectiveness of the oral contraceptive pill? − Gastrointestinal disorders − Antibiotics − Missed dose − **Season (time of year)**	Welcher Faktor beeinflusst in der Regel nicht die Wirksamkeit einer Verhütungspille? − Magen-Darm-Erkrankungen − Antibiotika − Vergessene Einnahme − **Jahreszeit**	0.88	0.32	0.26
HC_7	Which two hormones are found in most oral contraceptive pills (combined pills)? − **Progestin and estrogen** − Estrogen and testosterone − Progestin and luteinizing hormone (LH) − Adrenaline and estrogen	Welche zwei Hormone befinden sich in den meisten Verhütungspillenpräparaten (kombinierte Pille)? − **Gestagen und Östrogen** − Östrogen und Testosteron − Gestagen und luteinisierendes Hormon (LH) − Adrenalin und Östrogen	0.54	0.50	0.43
HC_8	What could be an advantage of hormonal contraceptives? − Concealment of relative energy deficit − **Relief of period (menstrual) pain** − Increased risk of thrombosis − No possibility to train according to menstrual cycle phases	Was könnte ein Vorteil hormoneller Verhütungsmittel sein? − Verschleierung des relativen Energiedefizites − **Linderung von Regelschmerzen** − Erhöhtes Thromboserisiko − Keine Möglichkeit des menstruationszyklusgesteuerten Trainings	0.82	0.38	0.34
HC_10	What can happen when taking the oral contraceptive pills as a hormonal contraceptive without a break (i.e., continuously)? − **The withdrawal bleed usually stops** − A woman becomes permanently infertile − The withdrawal bleed occurs continuously − The contraceptive pill as a contraceptive becomes ineffective	Was kann beim Einsatz der Pille als hormonelles Verhütungsmittel passieren, wenn diese im Langzeitzyklus (d.h. durchgehend) eingenommen wird? − **Die Blutung bleibt meistens aus** − Eine Frau bleibt über die Einnahme der Verhüttungspille hinaus unfruchtbar − Die Blutung tritt immer durchgehend auf − Die Verhütungspille wird als Verhütungsmittel unwirksam	0.67	0.47	0.26
HC_11	Which hormone(s) are contained in a hormonal IUS? − **Progestin only** − Estrogen only − Progestin and estrogen − Progestin and luteinizing hormone (LH)	Welches Hormon/ welche Hormone befinden sich in einer Hormonspirale? − **Nur Gestagen** − Nur Östrogen − Gestagen und Östrogen − Gestagen und luteinisierendes Hormon (LH)	0.08	0.27	0.28
HC_13	Which statement about the effect of the oral contraceptive pill (combined pill) is false? − It prevents ovulation − **It strengthens the woman's immunity so that her antibodies attack sperm** − It changes the lining of the uterus so that a fertilized egg is less likely to implant and survive − It thickens the mucus at the opening of the uterus (womb) so it is more difficult for sperm to penetrate	Welche Aussage über die Wirkung der Verhütungspille (kombinierte Pille) ist falsch? − Sie verhindert den Eisprung (Ovulation) − **Sie stärkt die Immunität einer Frau, sodass ihre Antikörper Spermien angreifen** − Sie verändert die Gebärmutterschleimhaut. sodass sich ein befruchtetes Ei weniger wahrscheinlich einnistet und überlebt − Sie verdickt den Schleim an der Öffnung der Gebärmutter (Uterus). sodass es für Spermien schwieriger ist einzudringen	0.45	0.50	0.27
HC_14	How does a hormonal IUS work? − It consistently prevents ovulation − It strengthens the woman's immunity so that her antibodies attack sperm − It thins the cervical mucus, making it difficult for sperm to enter the uterus − **It prevents the uterine lining from building up.**	Wie wirkt eine Hormonspirale? − Sie verhindert den Eisprung (Ovulation) − Sie stärkt die Immunität einer Frau. sodass ihre Antikörper Spermien angreifen − Sie verdünnt den Schleim an der Öffnung der Gebärmutter (Uterus). sodass es für Spermien schwieriger ist, einzudringen − **Der Schleimhautaufbau in der Gebärmutter (Uterus) wird verhindert**	0.21	0.40	0.21

The correct answer is in bold font. The answer format “Do not know” was added to each item.

*SD*, standard deviation; IUS, Intrauterine System.

In step 7 (construct validity of the HCKQ), knowledge differed significantly between participant groups (*F*(2,728) = 6.51, *p* = .002, *ηp^2^* = .02, *f* = .13, *n* = 731). One coach with unspecified sex was excluded from the sex-based group comparison. *Post-hoc* Bonferroni tests showed that knowledge scores among the female coaches (*M* = 5.45, *SD* = 1.87, *n* = 55) were significantly higher than for the male coaches (*M* = 4.27, *SD* = 1.96, *n* = 114; *p* = .001) and the female athletes (*M* = 4.58, *SD* = 2.03, *n* = 562; *p* = .007), while no significant differences were found between the male coaches and female athletes (*p* = .401). For the comparison between HC users and non-HC users, 18 female athletes were excluded because they reported not yet reaching menarche. Female athlete HC users had significantly higher knowledge scores (*M* = 5.02, *SD* = 1.86, *n* = 162) than non-HC users (*M* = 4.51, *SD* = 2.03, *n* = 382; *t*(330.24) = −2.84, *p* = .005, *r* = .15).

In Step 8 (internal consistency), a Cronbach's *α* of .61 was determined. This relatively low value can be explained by the fact that several items were retained for content validity despite not meeting the usual criteria for item difficulty or item-total correlation. Removing these items would not have improved the reliability coefficient. Additional reliability estimates provided similar results (McDonald's *ω* = .62; Guttman's *λ*₂ = .63).

In step 9 (test-retest reliability), knowledge scores were not significantly different between the two time points (*M* = 4.98, *SD* = 1.89 and *M* = 5.22, *SD* = 2.01, respectively; *p* = .185). The Bland-Altman analysis showed a mean difference of .23 points (95% limits of agreement ranging from .11 to .57), indicating no evidence of systematic bias in knowledge scores. No additional exposure to HC information was reported by 70% of the participants during the interval, while 30% reported only minimal exposure. The test-retest correlation was strong (Spearman's *ρ* = .75, *p* < .001) and the ICC indicated moderate reliability (ICC = .74, 95% CI = .61–.84).

## 4 Discussion

This study aimed to develop and validate the HCKQ, a tool designed to assess knowledge about HCs among female athletes and coaches across various sports. The questionnaire covered four topics: safety of HC use, functions and effects of different types of HCs (i.e., OCPs and IUSs), risk factors and side effects associated with HC use, and the fertile window within the menstrual cycle. The findings provide initial evidence supporting the content validity, known-groups validity, and reliability of HCKQ scores for assessing HC-related knowledge, suggesting that the HCKQ may be a useful tool for future research and practice.

The questionnaire development process combined expert input and a thorough review of the literature. Gynecologists contributed practical knowledge based on daily clinical experience, ensuring that the items reflected relevant and real-world questions. A comprehensive review of scientific and non-scientific sources informed the item pool. Previous studies by Johnson (3) and Larsen et al. (4) provided a foundation for specific items, with HC_10, HC_13, and HC_15 adopted directly, and items HC_07, HC_08, HC_09, HC_16, and HC_17 modified to align with a standardized response format (i.e., four answer options and a “do not know” option). This single-choice response format (as opposed to including questions with more than one correct answer) enhanced data processing efficiency and simplified the statistical analysis (46). This is a unique feature of the HCKQ, differing from the newly developed questionnaire by Larsen et al. (2025). Unlike existing instruments (e.g., 3, 4), the HCKQ includes items about contraceptive mechanisms, thus providing a deeper understanding of how different HC methods work. Such knowledge is essential for informed decision-making about HC use, particularly in elite sport where internal physiology (e.g., hormonal fluctuations) can influence physical responses (e.g., aerobic fitness, muscle strength, balance), psychological factors (e.g., mental sharpness), and sleep quality (17).

The HCKQ demonstrated a broad range of item difficulties, reflecting its ability to distinguish between individuals and groups with different levels of HC-related knowledge. Notably, we included items relating to IUSs, a method of contraception previously underrepresented in HC knowledge assessment tools (e.g., 3, 4). Given the wide range of HC types currently in use (12), which differ in composition (e.g., combined vs. progestin-only) and route of administration (oral, intrauterine, and implantable; 8, 9), a knowledge questionnaire needs to reflect this diversity. A robust understanding of HC types, mechanisms, and physiological effects is essential for female athletes to make informed decisions regarding health, well-being, and performance (4). In line with this, and similar to Larsen et al. (25), the HCKQ addresses various types of HCs, taking into account the range of available options and the different ways in which they work.

A multidisciplinary expert panel, including gynecologists, biologists, sports physicians, and sports scientists, supported the content relevance and clarity of the HCKQ. The HCKQ also distinguished between groups expected to differ in HC knowledge, with female coaches scoring higher than male coaches and female athletes. This finding is consistent with Johnson (3) and may reflect greater personal and professional exposure to reproductive health topics among female coaches. Female athletes often report feeling more comfortable discussing menstrual-cycle-related issues with female than male coaches, partly because female coaches are perceived as having greater personal understanding of these topics (47, 48). In contrast, male coaches may be less likely to be involved in such conversations because menstrual cycle and HC topics are often perceived as private or sensitive (10, 48, 49). Female athletes using HCs also scored higher than non-HC users, consistent with Larsen et al. (4), which may reflect greater exposure to HC-related information through personal use, medical counseling, prescription processes, or information seeking. This interpretation is supported by evidence showing higher OCP knowledge among users than non-users in non-athlete samples (50). However, the effect sizes were small in our study, indicating that group membership and HC use explained only limited differences in HC knowledge; therefore, the practical significance of these findings should be interpreted cautiously.

Reliability of the HCKQ was assessed using several methods. Internal consistency was modest, with Cronbach's *α* = .61, McDonald's *ω* = .62 and Guttman's *λ*₂ = .63. This pattern of results suggests that Cronbach's *α* may have underestimated the scale's true reliability due to violations of the tau-equivalence assumption (51). Unlike previous HC questionnaires (3, 25), the present study reports multiple forms of internal reliability, highlighting a broader approach to psychometric evaluation. Nevertheless, Cronbach's *α* remains the most widely reported indicator, allowing for direct comparisons with other instruments. Cronbach's *α* reflects how closely related the items are, and the extent to which the items assess the same underlying concept (in this case, HC-related knowledge; 27). However, Cronbach's *α* typically increases with the number of test items (27). Given that the HCKQ includes only 10 items covering multiple topics, the relatively low internal consistency in the present study may reflect greater content diversity spread across fewer items, rather than a weakness of the instrument. Additionally, several items showed low item-total correlation but were retained because they addressed critical concepts, supporting content validity despite weaker statistical homogeneity. By comparison, Johnson's (3) HC questionnaire achieved a Cronbach's *α* of .84 but included 12 items, used multiple-choice answers, and focused primarily on OCPs. More recently, Larsen et al. (25) reported an overall Cronbach's *α* of .93, including 46 items across four menstrual cycle and HC knowledge domains. This would imply different applications of our 10-item HCKQ compared to Larsen et al.'s (25) 46-item questionnaire. The moderate Cronbach's *α* measured in the current study reflects limited internal consistency of the HCKQ, which must be considered when interpreting results and determining the practical utility of the tool.

Test-retest reliability was evaluated using responses from sport science students at two time points. Results showed minimal discrepancies between tests, with a mean score increase of .23 points, suggesting a minor learning effect. In addition to correlation analysis, a Bland-Altman plot indicated strong agreement between the two test scores, with slightly greater variability for lower scores. These findings indicate that the HCKQ demonstrates moderate reliability, as reflected in the Spearman's correlation and ICCs. The questionnaire provides a foundation for further refinement and can be applied in both research and practical settings to assess HC-related knowledge among female athletes and coaches.

### 4.1 Limitations and future applications

In this study, the female athletes and coaches were grouped together for item analyses in steps 4 and 6. This approach did not account for potential subgroup differences, which is a limitation, particularly since athletes made up the majority of respondents (88% in step 4; 77% in step 6). As a result, item selection may have been biased towards female athletes’ knowledge profiles, and separate analyses of female athletes, female coaches, and male coaches might have led to different item-selection decisions. Another limitation is that some subgroups in step 7 were slightly smaller than the ideal sample size calculated (e.g., HC users: *n* = 162 instead of > 196; female coaches: *n* = 55 instead of > 59), which may have reduced the statistical power of the subgroup comparisons. Although statistically significant differences were observed between female and male coaches and between HC users and non-HC users, the associated effect sizes were small. These findings should therefore be interpreted cautiously. Regarding test-retest reliability, temporal stability was assessed in sport science students. This population was selected because of their sport-related educational background and accessibility. However, they may differ from athletes and coaches in HC knowledge, survey familiarity, and response behavior. Therefore, future studies should confirm test-retest reliability of the HCKQ in samples relevant to the population(s) of interest.

HC_11 was answered correctly by only 8% of participants. Its low difficulty index suggests that the item may be too difficult or insufficiently tailored to the current population, potentially limiting its contribution to score discrimination. Future revisions of the HCKQ should therefore review HC_11 for potential rewording, simplification, or replacement, and subsequently re-evaluate its item difficulty and discrimination. Also of note is that the HCKQ was validated in German, and the items were translated into English for the purpose of international publication. Future research should evaluate the questionnaire's validity and reliability in other languages and cultural contexts. Although the tool was developed within a sports setting, its content is suitable for use in non-sport populations. The HCKQ may serve as a research instrument to explore relationships between HC knowledge and other factors (e.g., demographics, perceived performance changes during a cycle, physiological and cognitive characteristics), as well as a screening tool to identify individuals requiring additional education. Pre- and post-intervention assessments using the HCKQ could help to evaluate and refine HC education programs, thus supporting the development of high-quality educational resources on HC knowledge both within and beyond sport. In practical settings, the HCKQ may be used as a screening tool to identify knowledge gaps to guide education or counseling conversations. However, it should not be used as a stand-alone decision-making tool, as individualized HC decisions require consideration of medical history, personal preferences, symptoms, training demands, and performance-related goals in consultation with qualified healthcare professionals.

## 5 Conclusion

The 10-item HCKQ provides a useful tool for assessing HC-related knowledge among female athletes and coaches, with evidence of content validity and moderate reliability. The questionnaire covers four essential topics: safety of HC use, function and effects of different types of HCs, risk factors and side effects associated with HC use, and the fertile window within the menstrual cycle. Translating and validating the HCKQ in other languages is an important next step to enable wider use. Beyond measurement, the HCKQ can help identify knowledge gaps, facilitate open discussions about reproductive health and HCs, and guide educational initiatives for athletes, coaches, and support staff. By promoting informed dialogue, the HCKQ has the potential to enhance understanding and collaboration regarding HC use and effects in sport environments, and beyond.

## Data Availability

The raw data supporting the conclusions of this article will be made available by the authors, without undue reservation.
