# Supplementary material for: The development and validation of a questionnaire to assess female athletes’ and coaches’ knowledge about hormonal contraception
Source: Front Sports Act Living. 2026 Oct 23;8:1921305. doi: 10.3389/fspor.2026.1921305 (PMC13739691; doi:10.3389/fspor.2026.1921305)
Supplement: Supplementary file 1 [file Table1.docx]

Supplementary Material

**Supplementary Table 1:** An overview of the items included in the initial development of the hormonal contraception knowledge questionnaire (Pre-HCKQ) for female athletes and coaches (green text has been translated from German)

| Item no. | Gynecologist 1 | Gynecologist 2 | Gynecologist 3 | Research of scientific and non-scientific questionnaires | Initial Item |
| --- | --- | --- | --- | --- | --- |
| HC_1 | When are the fertile days? |  |  | On which days between two menses are you most prone to become pregnant?  a) At the beginning  b) At the middle  c) At the end  d) Whenever (Szücz et al., 2016) | When are the fertile days in a menstrual cycle?   - **Middle of the menstrual cycle** - During menstruation - At the end of the menstrual cycle - All days are equally fertile |
| HC_2 | How long are sperm viable? |  |  | How long can sperm remain fertile in a woman's body?  a) 3 minutes  b) 24 hours  c) 2 to 6 days  d) 3 to 5 days (Krahlisch, 2013) | How long are sperm viable?   - 12 hours - 1 day - **3-4 days** - 5-8 days |
| HC_3 | Which method of contraception is safer, the condom or the pill? |  | Name at least four different hormonal contraceptive methods. | Which of the contraceptive methods listed below are characterized by the highest effectiveness?  a) Natural contraceptive methods/ family planning (daily readings of the body temperature, observation of cervical mucus).  b) Oral hormonal contraception.  c) Emergency (postcoital) contraception.  d) Hormonal contraception as implants, patches.  e) Spermicides.  f) Barrier contraception (condom/vaginal cap).  g) Intrauterine devices. (Warzecha et al., 2019) Which of the following is an effective form of birth control?  a) condoms  b) cervical caps  c) IUDs  d) all of the above (Dobrogosz, 2020) | Which of the following contraceptive methods is the safest?   - **Pill** - Condom - Temperature measurement - Calendar method |
| HC_4 | How long is an egg ready for fertilization? |  |  | How many days is a woman fertile in her cycle?  a) one  b) three  c) eight  d) twelve (Trackle GmbH, n.d.)  Approximately how long after ovulation does the egg survive?  a) 4 days  b) 12 hours  c) 24 hours (Potter, 2011) | How long is an egg fertile?   - 8 hours - **1 day** - 3-4 days - 5-8 days |
| HC_5 |  | What does the PEARL index represent? | What does the PEARL index represent? |  | What is the PEARL index?   - Measurement of a woman's fertility - Measurement of how many menstrual cycles a woman will have in her lifetime - **Measurement of the reliability of a contraceptive method** - Measurement of the composition of hormones in the contraceptive pill |
| HC_6 |  |  |  |  | What does not influence the effect of the pill?   - Gastrointestinal diseases - Antibiotics - Traveling with a time difference - **Season** |
| HC_7 |  | What does the micro pill consist of? | What are the two hormones that are found in most birth control pills? | One type of oral contraception is called the combined pill. How many hormones are in this form of contraception? (Larsen et al., 2020) | Which two hormones are in most birth control pills?   - **Estrogen and progestin** - Estrogen and testosterone - Progestogen and LH - Adrenaline and estrogen |
| HC_8 |  | What are the advantages of taking the pill?  What are the benefits of an IUS? | Please name at least three advantages of hormonal contraceptive methods. | Circle all of the benefits you believe can potentially result from the use of birth control pills.  a) menstrual cycle regulation  b) relief from headaches  c) relief from painful periods  d) relief from PMS  e) decreased menstrual blood flow  f) reduced risk for endometrial cancer  g) reduced risk for ovarian cancer  h) reduce risk of breast disease  i) reduced risk of pelvic inflammatory disease  j) reduced risk of sexually transmitted diseases  k) reduced acne  l) reduced iron-deficiency anemia  m) fewer ovarian cysts  n) fewer ectopic pregnancies  o) I am not sure (Johnson, 2008) | What is not an advantage of hormonal contraceptive methods?   - Safe contraceptive method when used correctly - Reduction of menstrual pain - Improved skin texture - **Improved lactose tolerance (lactose intolerance)** |
| HC_9 | Does the pill increase the risk of thrombosis? | What are the side effects of the pill?  Does the contraceptive pill cause you to get fat? | What are the most common side effects associated with the pill? Name at least 3! | Circle all of the risks you believe are potentially associated with the use of birth control pills.  a) bleeding irregularities  b) headache or migraine  c) gastrointestinal upset  d) breast tenderness  e) weight gain  f) blood clotting  g) acne  h) hypertension  i) increased risk for human papillomavirus  j) increased risk for cervical cancer  k) fatigue  l) I am not sure (Johnson, 2008) | What are the common side effects of the pill?   - Change in the desire for sex (libido) - Breast tenderness - Increased risk of thrombosis - **All of the above** |
| HC_10 | Can pills be taken without interruption? |  | Can I change the pill break if I take a combined oral contraceptive pill (2-hormone pill)? | What may happen when "inactive" pills are skipped and "active" pills are continuously taken in their place?  a) menstrual periods stop  b) a woman becomes permanently infertile  c) menstrual periods occur continuously  d) the pill as a contraceptive becomes ineffective  e) I am not sure (Johnson, 2008)  What may happen when 'inactive' pills are skipped and 'active' pills are continuously taken in their place?  a) menstrual periods stop  b) a woman becomes permanently infertile  c) menstrual periods occur continuously  d) the pill as a contraceptive becomes ineffective  e) I am not sure (Larsen et al., 2020)  Suppressing monthly bleeding or 'skipping your period' via contraception is unhealthy.  a) True  b) False (ABC News, 2017) | What can happen when taking the pill as a hormonal contraceptive without a break (i.e., continuously)?   - The withdrawal bleed usually stops - A woman becomes permanently infertile - The withdrawal bleed occurs continuously - The contraceptive pill, as a contraceptive, becomes ineffective |
| HC_11 |  | How does it differ from a hormonal IUS? |  |  | Which hormone(s) are contained in a hormonal IUS?   - Progestin only - Estrogen only - Estrogen and progestin - Progestin and luteinizing hormone (LH) |
| HC_12 | How long does a hormonal IUS last? | How long can a hormonal IUS stay in the uterus? | How often should a hormonal IUS be replaced? |  | How long does a hormonal IUS work on average?   - 1 year - **3-5 years** - 5-7 years - For life |
| HC_13 |  | What does the pill do? | How do the pill and most hormonal contraceptive methods work? | Which of the following is not a way in which the combined pill works to prevent pregnancy?  a) It prevents the ovaries from releasing eggs  b) It boosts a woman's immunity so that her antibodies attack sperm  c) it changes the lining of the uterus so a fertilized egg is less likely to implant and survive  d) It thickens mucus at the opening of the uterus, so it is harder for sperm to penetrate  e) I am not sure (Larsen et al., 2020) | Which statement about the effect of the pill is false?   - It prevents ovulation - **It strengthens the woman's immunity so that her antibodies attack sperm** - It changes the lining of the uterus so that a fertilized egg is less likely to implant and survive - It thickens the mucus at the opening of the uterus (womb), so it is more difficult for sperm to penetrate |
| HC_14 | Does the hormonal IUS prevent ovulation? | How does a hormonal IUS work? |  |  | How does a hormonal IUS work?   - **The build-up of the mucous membrane in the uterus is prevented** - It prevents ovulation - It strengthens the woman's immunity so that her antibodies attack sperm - It thins the cervical mucus, making it difficult for sperm to enter the uterus. |
| HC_15 |  |  |  | What medical condition(s) may make it dangerous for a woman to use the pill?  a) high blood pressure  b) smoking  c) diabetes  d) none of the above  e) all of the above  f) I am not sure (Johnson, 2008) | What is a risk factor for taking the pill?   - High blood pressure - Smoking - Diabetes - **All of the above** |
| HC_16 |  |  | What distinguishes the minipill from other monophasic and multiphasic preparations? | Another type of oral contraception is called the mini pill. How many hormones are in this form of contraception? (Larsen et al., 2020)  Which hormonal active ingredient is in the mini pill?  a) Estrogen  b) both  c) Progestin (Eltern, n.d.)  There are different formulas of the combined pill, called monophasic, biphasic, and triphasic. Were you aware of the three different combined pill formulas stated above prior to today?  a) yes  b) no (Larsen et al., 2020) | What distinguishes the minipill from other monophasic and multiphasic birth control pills?   - Only contains progestin - Strict adherence to the intake times is necessary - Continuous intake is absolutely necessary - **All of the above** |
| HC_17 | Which is more reliable for contraception: condoms or the pill? | What other methods of contraception are there? | Categorize these contraceptive methods according to their anti-conceptual safety (start with the least safe): Temperature measurement, pill, IUS, condom, diaphragm! | When taken properly, how effective is the combined oral contraceptive pill at preventing pregnancy? Please write your answer as a percentage:  (Larsen et al., 2020) | Which classification of the effectiveness of different contraceptives (starting with the least safe) is correct?   - **Temperature measurement < diaphragm < condom < pill < coil** - Temperature measurement < Pill < IUS < Diaphragm < Condom - Condom < IUS < Temperature measurement < Diaphragm < Pill - Diaphragm < Temperature measurement < Condom < IUS < Pill |

*Notes*: The correct answer is marked in bold. The "Do not know" answer format was added to each item.

*Abbreviations:* IUS = Intrauterine System

**References**

ABC News. (2017, October 5). *Period quiz: Do you know menstruation fact from fiction? ABC News.* https://www.abc.net.au/news/health/2017-10-05/do-you-know-menstruation-fact-from-fiction/9010430

Dobrogosz, H. (2020, November 9). Can You Pass A Middle School Sex Ed Test? BuzzFeed. Retrieved from https://www.buzzfeed.com/hannahdobro/can-you-pass-a-middle-school-sex-ed-test. [Accessed on June 19, 2024]

Eltern. (n.d.). *Quiz: Kennen Sie Ihren Zyklus?* Eltern. https://www.eltern.de/node/10706/take

Johnson, T. R. (2008). *Knowledge and Attitudes Regarding the Menstrual Cycle, Oral Contraceptives, and Sport Performance: The Conceptualization and Development of a Questionnaire for Athletic Coaches* [Dissertation]. Florida State University Libraries, Florida.

Krahlisch, M. (2013, July 1). Menstruationszyklus-Quiz - Teste Dich. Retrieved from https://www.testedich.de/gesundheitstests/verhuetung-kinderwunsch-schwangerschaft/periode-regel-tage/quiz32/1372064207/menstruationszyklus-quiz. [Accessed on June 6, 2024]

Larsen, B., Morris, K., Quinn, K., Osborne, M., & Minahan, C. (2020). Practice does not make perfect: A brief view of athletes' knowledge on the menstrual cycle and oral contraceptives. *Journal of Science and Medicine in Sport, 23*(8), 690–694. https://doi.org/10.1016/j.jsams.2020.02.003

Potter, L. (2011, June 28). Menstrual Cycle Quiz. Retrieved from geekymedics.com/menstrual-cycle-quiz. [Accessed on June 6, 2024]

Szűcs, M., Bitó, T., Csíkos, C., Párducz Szöllősi, A., Furau, C., Blidaru, I., Kapamadzija, A., Sedlecky, K., & Bártfai, G. (2017). Knowledge and attitudes of female university students on menstrual cycle and contraception. *Journal of Obstetrics and Gynaecology, 37*(2), 210–214. doi:10.1080/01443615.2016.1229279.

Trackle GmbH. (n.d). Zyklus Quiz. Retrieved from https://trackle.de/zyklus/zyklus-quiz/. [Accessed on June 6, 2024]

Warzecha, D., Szymusik, I., Pietrzak, B., Kosinska-Kaczynska, K., Sierdzinski, J., Sochacki-Wojcicka, N., & Wielgos, M. (2019). Sex education in Poland - a cross-sectional study evaluating over twenty thousand Polish women's knowledge of reproductive health issues and contraceptive methods. *BMC Public Health, 19*(1), 689. doi:10.1186/s12889-019-7046-0.

**Supplementary Table 2:** Relevance and clarity for each item in the 10-item hormonal contraception knowledge questionnaire (HCKQ), as rated by the expert panel (*n* = 18)

|  | Relevance | | | | | Clarity | | | | |
| --- | --- | --- | --- | --- | --- | --- | --- | --- | --- | --- |
|  | *M* | *SD* | *Median* | *min* | *max* | *M* | *SD* | *Median* | *min* | *max* |
| HC_3 | 5.7 | 0.6 | 6 | 4 | 6 | 5.4 | 1.0 | 6 | 3 | 6 |
| HC_4 | 5.4 | 0.9 | 6 | 3 | 6 | 5.6 | 0.6 | 6 | 4 | 6 |
| HC_5 | 5.3 | 1.1 | 6 | 2 | 6 | 5.7 | 0.8 | 6 | 3 | 6 |
| HC_6 | 5.4 | 1.0 | 6 | 3 | 6 | 5.2 | 1.2 | 6 | 2 | 6 |
| HC_7 | 5.6 | 0.7 | 6 | 4 | 6 | 5.8 | 0.5 | 6 | 4 | 6 |
| HC_8 | 5.4 | 0.8 | 6 | 4 | 6 | 4.9 | 1.1 | 5 | 3 | 6 |
| HC_10 | 5.6 | 0.7 | 6 | 4 | 6 | 5.5 | 0.8 | 6 | 3 | 6 |
| HC_11 | 5.2 | 0.9 | 6 | 3 | 6 | 5.7 | 0.7 | 6 | 4 | 6 |
| HC_13 | 5.5 | 0.7 | 6 | 4 | 6 | 5.1 | 1.1 | 5 | 3 | 6 |
| HC_14 | 5.6 | 0.7 | 6 | 4 | 6 | 5.8 | 0.5 | 6 | 4 | 6 |
| **Total** | **5.5** | **0.8** | **6** | **2** | **6** | **5.5** | **0.9** | **6** | **2** | **6** |

*Notes*: *M* = mean value, *SD* = standard deviation, *Min* = minimum, *Max* = maximum. Relevance: 1 = not at all relevant, 6 = very relevant. Clarity: 1 = not at all clear, 6 = very clear.

**Supplementary Table 3:** Item descriptions (English translations) for the final 10-item hormonal contraception knowledge questionnaire (HCKQ), including comments from the expert panel (*n* = 18) (questionnaire and video conference)

|  | Comments (questionnaire/video conference) | Item (translated in English) |
| --- | --- | --- |
| HC_3 | Oral contraceptive pill  Add to question: most effective according to the PEARL index | Which of the following contraceptive methods is the most effective according to the PEARL index?   - Oral contraceptive pill - Condom - **Hormonal IUS** - Calendar method |
| HC_4 | Risk of confusion with fertile days, rephrase question -> Sperm can survive up to 5 days in the female genital tract. How long is an egg fertile? | Sperm can survive for up to 5 days in the female genital tract. How long is an egg fertile?   - approx. 8 hours - **approx. 1 day** - approx. 3–4 days - approx. 5–8 days |
| HC_5 |  | What is the PEARL index?   - Measurement of a woman's fertility - Measurement of how many menstrual cycles a woman will have in her lifetime - **Measurement of the reliability of a contraceptive method** - Measurement of the composition of hormones in the contraceptive pill |
| HC_6 | Which factor does not generally influence the effectiveness of an oral contraceptive pill? | Which factor does not generally influence the effectiveness of the oral contraceptive pill?   - Gastrointestinal disorders - Antibiotics - Missed dose - **Season (time of year)** |
| HC_7 | Oral contraceptive pill (combined pill)  Write out LH | Which two hormones are found in most oral contraceptive pills (combined pills)?   - **Estrogen and progestin** - Estrogen and testosterone - Progestin and luteinizing hormone (LH) - Adrenaline and estrogen |
| HC_8 | What is not a disadvantage? (more clearly that the other three answers are not wrong)  Answer 3 can be rated positively:  increased risk of thrombosis  Answer 1 unclear | What could be an advantage of hormonal contraceptives?   - Concealment of relative energy deficit - **Relief of period (menstrual) pain** - Increased risk of thrombosis - No possibility to train according to menstrual cycle phases |
| HC_10 | Rephrase answer 1:  The withdrawal bleed usually stops. Intermittent bleeding/spotting possible  Rephrase answer 2:  A woman becomes permanently infertile. | What can happen when taking the oral contraceptive pills as a hormonal contraceptive without a break (i.e., continuously)?   - **The withdrawal bleed usually stops** - A woman becomes permanently infertile - The withdrawal bleed occurs continuously - The contraceptive pill, as a contraceptive, becomes ineffective |
| HC_11 |  | Which hormone(s) are contained in a hormonal IUS?   - **Progestin only** - Estrogen only - Estrogen and progestin - Progestin and luteinizing hormone (LH) |
| HC_13 | (combined pill)  Please note: progestogen-only mini-pills such as Desogestrel 75 µg usually do NOT prevent ovulation. A combined pill (estrogen and progestin) would be correct here.  The mini pill does not necessarily prevent ovulation. | Which statement about the effect of the oral contraceptive pill (combined pill) is false?   - It prevents ovulation - **It strengthens the woman's immunity so that her antibodies attack sperm** - It changes the lining of the uterus so that a fertilized egg is less likely to implant and survive - It thickens the mucus at the opening of the uterus (womb), so it is more difficult for sperm to penetrate |
| HC_14 |  | How does a hormonal IUS work?   - It consistently prevents ovulation - It strengthens the woman's immunity so that her antibodies attack sperm - It thins the cervical mucus, making it difficult for sperm to enter the uterus - **It prevents the uterine lining from building up.** |

*Notes*: The correct answer is marked in bold. The answer format “Do not know” was added to each item.
